# Interferon response and profiling of interferon response genes in peripheral blood of vaccine-naive COVID-19 patients

**DOI:** 10.3389/fimmu.2023.1315602

**Published:** 2024-01-10

**Authors:** Baozhen Huang, Jinghan Huang, Nim Hang Chiang, Zigui Chen, Grace Lui, Lowell Ling, Mike Yat Wah Kwan, Joshua Sung Chih Wong, Phoebe Qiaozhen Mak, Janet Wan Hei Ling, Ivan Cheuk San Lam, Rita Wai Yin Ng, Xingyan Wang, Ruonan Gao, David Shu-Cheong Hui, Suk Ling Ma, Paul K. S. Chan, Nelson Leung Sang Tang

**Affiliations:** ^1^ Department of Chemical Pathology, and Li Ka Shing Institute of Health Science, Faculty of Medicine, The Chinese University of Hong Kong, Hong Kong, Hong Kong SAR, China; ^2^ Department of Microbiology, The Chinese University of Hong Kong, Hong Kong, Hong Kong SAR, China; ^3^ Department of Medicine and Therapeutics, Faculty of Medicine, The Chinese University of Hong Kong, Hong Kong, Hong Kong SAR, China; ^4^ Department of Anaesthesia and Intensive Care, The Chinese University of Hong Kong, Hong Kong, Hong Kong SAR, China; ^5^ Paediatric Infectious Disease Unit, Department of Paediatrics and Adolescent Medicine, Princess Margaret Hospital, Hong Kong, Hong Kong SAR, China; ^6^ Department of Psychiatry, The Chinese University of Hong Kong, Hong Kong, Hong Kong SAR, China; ^7^ Hong Kong Branch of CAS Center for Excellence in Animal Evolution and Genetics and KIZ/CUHK Joint Laboratory of Bioresources and Molecular Research in Common Diseases, Hong Kong, Hong Kong SAR, China; ^8^ Functional Genomics and Biostatistical Computing Laboratory, CUHK Shenzhen Research Institute, Shenzhen, China

**Keywords:** COVID-19, IFN, ISGs, biomarker, severity

## Abstract

**Introduction:**

There is insufficient understanding on systemic interferon (IFN) responses during COVID-19 infection. Early reports indicated that interferon responses were suppressed by the coronavirus (SARS-CoV-2) and clinical trials of administration of various kinds of interferons had been disappointing. Expression of interferon-stimulated genes (ISGs) in peripheral blood (better known as interferon score) has been a well-established bioassay marker of systemic IFN responses in autoimmune diseases. Therefore, with archival samples of a cohort of COVID-19 patients collected before the availability of vaccination, we aimed to better understand this innate immune response by studying the IFN score and related ISGs expression in bulk and single cell RNAs sequencing expression datasets.

**Methods:**

In this study, we recruited 105 patients with COVID-19 and 30 healthy controls in Hong Kong. Clinical risk factors, disease course, and blood sampling times were recovered. Based on a set of five commonly used ISGs (IFIT1, IFIT2, IFI27, SIGLEC1, IFI44L), the IFN score was determined in blood leukocytes collected within 10 days after onset. The analysis was confined to those blood samples collected within 10 days after disease onset. Additional public datasets of bulk gene and single cell RNA sequencing of blood samples were used for the validation of IFN score results.

**Results:**

Compared to the healthy controls, we showed that ISGs expression and IFN score were significantly increased during the first 10 days after COVID infection in majority of patients (71%). Among those low IFN responders, they were more commonly asymptomatic patients (71% vs 25%). 22 patients did not mount an overall significant IFN response and were classified as low IFN responders (IFN score < 1). However, early IFN score or ISGs level was not a prognostic biomarker and could not predict subsequent disease severity. Both IFI27 and SIGLEC1 were monocyte-predominant expressing ISGs and IFI27 were activated even among those low IFN responders as defined by IFN score. In conclusion, a substantial IFN response was documented in this cohort of COVID-19 patients who experience a natural infection before the vaccination era. Like innate immunity towards other virus, the ISGs activation was observed largely during the early course of infection (before day 10). Single-cell RNA sequencing data suggested monocytes were the cell-type that primarily accounted for the activation of two highly responsive ISGs (IFI44L and IFI27).

**Discussion:**

As sampling time and age were two major confounders of ISG expression, they may account for contradicting observations among previous studies. On the other hand, the IFN score was not associated with the severity of the disease.

## 1 Introduction

The current pandemic coronavirus disease 2019 (COVID-19) is caused by the infection of the severe acute respiratory syndrome coronavirus 2 (SARS-CoV-2). The clinical presentations are highly variable, ranging from asymptomatic to severe disease leading to intensive care admission and even death. Both innate and adaptive immune response play important roles in both defenses against the virus and disease course.

Interferons (IFN) play a fundamental role in the innate immune system. They act as inhibitors of viral replication in infected cells and have a defensive action in uninfected cells. However, some studies suggested that delayed IFN-I response and lower levels of IFN-I and -III are special features after SARS-CoV-2 infection, both in cell line and animal models (1, 2). Furthermore, soon after the outbreak of COVID-19, several studies reported unexpectedly low IFN-I levels in COVID-19 patients (3, 4) supporting the notion that IFN response was impaired in some patients (5, 6). Studies also suggested that IFN-I increased with tumor necrosis factor (TNF) and interleukin-1 (IL-1) in severe cases but not in mild cases (7). Additionally, a clinical study carried out early during the pandemic suggested that administration of IFN was beneficial in viral clearance and reduction of inflammatory cytokines (8). All these data provide support for treatment with exogeneous interferon in COVID-19 patients.

However, other COVID-19 studies observed a substantial IFN response. For example, in studies measuring interferon stimulated genes (ISGs) in bronchoalveolar lavage fluid (BALF, containing both cells of the respiratory epithelium and immune cells), SARS-CoV-2 robustly triggered the expression of numerous ISGs (9, 10). ISGs were increased in the T cell and monocytes of critical COVID-19 patients (6) and also in the PBMCs of moderate severity patients (11). These results are in contrast with other studies reporting dysfunction of IFN caused by SAR-CoV-2, for example Lee et al. reported dysregulation of IFN-I activity and inflammation were the cause of severe COVID-19 (5). It is uncertain if ISGs or IFN response is impaired in leukocytes after infection.

The first-ever global scale vaccination against SARS-CoV-2 started from the end of 2020 represents a major achievement of public health in the history of mankind (12, 13). Vaccination activated the essential acquired immunity to reduce disease sequel of the infection. After the introduction of vaccination, natural immune response against infection is changed. Therefore, it is expected the immune response of patients with (predominant adaptive immunity) or without (innate immunity) prior vaccination are different. In order to understand the natural innate immune response to SARS-CoV-2, study of patients without prior vaccination is required as it cannot be revealed with patients with vaccination history. So that, archival samples of vaccine-naïve COVID-19 patients taken before the era of vaccination are most valuable in this regard. Here, we retrieved archival blood samples from a small cohort of patients before vaccination that were available to study the interferon response of a natural COVID-19 infection.

While early trial results of recombinant interferon treatment had been controversial, the results of subsequent worldwide solidarity trials conducted by WHO demonstrated that interferon regimens appeared to have little or no effect on the outcome of hospitalized COVID-19 patients (14). This was also supported by the Adaptive COVID-19 Treatment Trial 3 (ACTT-3), which showed no benefit of treatment with interferon beta-1a together with antiviral remdesivir than treatment with just remdesivir in hospitalized patients (15). However, research of early treatment Pegylated Interferon Lambda for COVID-19 patients who had been vaccinated, reported more rapid viral clearance and reduced risk of requiring hospitalization (16).

These contradictory findings may underline a complex interaction between the SAR-CoV-2 virus and the host’s IFN response (17). On one hand, inhibition of IFN production was observed in SARS-CoV and Middle East respiratory syndrome (MERS)-CoV (18, 19). Other studies showed that ISG expression was markedly increased in the upper respiratory tract in patients with COVID-19 (20, 21). Few studies examined specifically early IFN response in leukocytes in COVID patients. As these studies did not explicitly investigate the time course of the IFN response, it is unclear if the sampling time, tissue type (respiratory epithelium versus blood leukocytes) and the definition of disease severity caused contradictory findings on the IFN response in patients with COVID-19.

Interferon represents an early host response, early sample collection (within 10 days after onset) was particularly difficult but they are essential for a better understanding of the role of IFN response in COVID-19. In this regard, we try to address several research questions with this study. Is there an inadequate IFN response within the first 10 days after onset of COVID-19 infection and does it predict the subsequent disease course and severity? We conducted this study with patients recruited in the first wave of COVID outbreak in Hong Kong to answer these research questions.

## 2 Materials and methods

### 2.1 Patients in this cohort and samples

**Figure 1:**
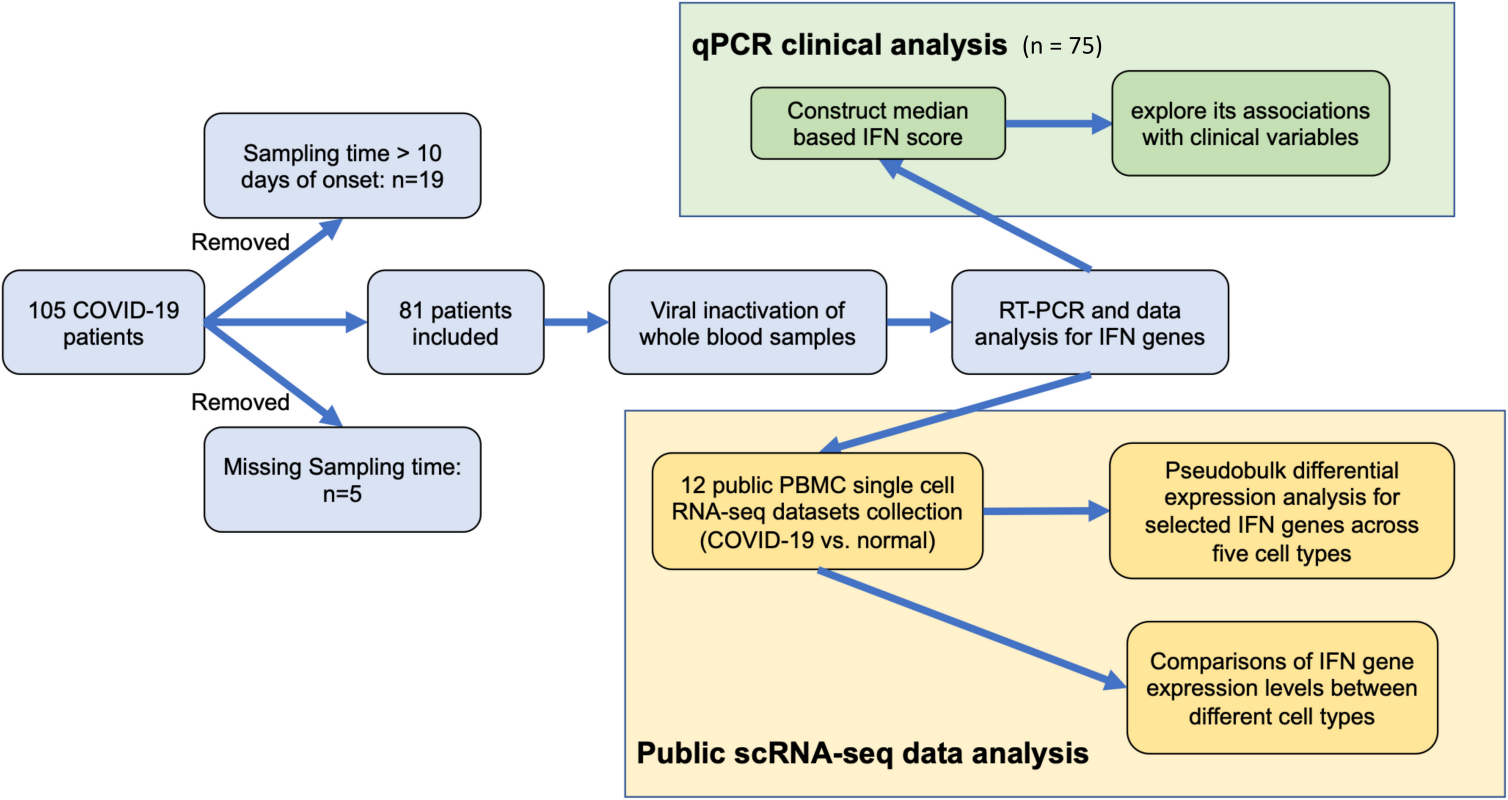
Study workflow.

From June 2020 to September 2020 before the availability of vaccine, a total of 81 COVID-19 patients with positive SARS-COV-2 testing results in public hospitals in Hong Kong were invited to join this study and their blood samples were collected within 10 days after disease onset and clinical data available (**Figure 1** and **Table 1**). Study approval was obtained from The Joint Chinese University of Hong Kong – New Territories East Cluster Clinical Research Ethics Committee (The Joint CUHK-NTEC CREC 2020.076). After receiving informed consent, 6 ml EDTA peripheral blood was collected for various investigation. 0.3 ml of whole blood was thoroughly mixed with 3x volume of TRIzol-BD (T3809, Sigma-Aldrich) to stabilize RNA in blood leukocytes and kept at -70C until total RNA extraction.

**Table 1:** Basic characteristics and general information of the cohort.

Characteristics	All (n=81)	Non-severe (n=68, 84%)	Severe (n=13, 16%)	P value^a^
**Age (years), mean (SD)**	39.95 (24.72)	34.54 (23.09)	68.23 (8.07)	<0.001^b^
**Day from onset, median (Q1-Q3)**	5 (3-7)	3.5 (2-7)	7 (5-8)	0.01^c^
**Day from admission, median (Q1-Q3)**	3 (1-4)	2 (1-4)	4 (3-6)	0.02^c^
**Female, n (%)**	39 (48.15%)	37 (54.41%)	2 (15.38%)	0.02^d^
**Current Smoker, n (%)**	13 (24.07%)	0 (0.00%)	13 (100%)	<0.001^d^
**IFN Treatment, n (%)**	29 (35.80%)	16 (23.53%)	13 (100%)	<0.001^d^
**CRP (mg/L), median (Q1-Q3)**	5.5 (0.83-27.13)	4.5 (0.65-14.88)	39.2 (13.13-73.13)	0.01^c^
**Peak viral load, median (Q1-Q3)**	4.85×10^7^ (3.28×10^5^-1.54×10^9^)	2.2410^7^ (2.5310^5^-1.6610^9^)	2.7710^8^ (3.6610^7^-1.4710^9^)	0.24^c^
**CBC-platelet (billion/L), median (Q1-Q3)**	204.5 (155.8-263.8)	218 (170.75-270.25)	150 (114.25-164.5)	0.002^c^
**WBC (10^9^/L), median (Q1-Q3)**	4.90 (3.80-6.55)	4.90 (4.08-6.13)	4.95 (3.40-9.13)	0.99^c^
**LYM%, median (Q1-Q3)**	27.65 (18.75-37.70)	32.75 (21.00-40.00)	11.50 (9.50-22.50)	<0.001^c^
**MON%, median (Q1-Q3)**	10.00 (7.98-13.93)	10.00 (8.00-13.23)	8.50 (6.00-14.75)	0.58^c^
**NEU%, median (Q1-Q3)**	57.75 (47.00-70.25)	56.00 (46.85-65.25)	77.00 (68.25-82.25)	<0.001^c^
**EOS%, median (Q1-Q3)**	0.90 (0-2)	1 (0-2)	0 (0-0)	0.01^c^
**BAS%, median (Q1-Q3)**	0.30 (0-1)	0.50 (0-1)	0 (0-0)	0.01^c^
**Neutrophil to Lymphocyte ratio, median (Q1-Q3)**	2.09 (1.31-3.63)	1.69 (1.20-3.16)	6.60 (2.90-8.88)	<0.001^c^
**Platelet to Lymphocyte ratio, median (Q1-Q3)**	0.26 (0.16-0.74)	0.24 (0.16-0.61)	0.37 (0.20-0.88)	0.48^c^

A total of 81 subjects in the cohort were divided into mild and severe COVID-19 cases and compared using statistical tests. T-test was used for continuous normally distributed variables and Mann Whitney U test was used for variables with skewed distribution. χ^2^ test was used for categorical variables. P values indicating statistical significance are shown.

IFN, Interferon; CRP, C-reactive protein; Q1-Q3, Quartile 1-3; CBC, Complete blood count; WBC, White blood cell; LYM, Lymphocyte; MON, Monocyte; NEU, neutrophil; EOS, Eosinophil; BAS, Basophil.

a. P value for the comparison between the two groups.

b. P value from T-test.

c. P value from rank sum test.

d. P value from χ^2^ test.

The disease course of COVID-19 infection was classified according to the WHO clinical progression scale with the broad classification into 4 groups: namely ambulatory mild, hospitalized moderate, hospitalized severe diseases and dead (22). A few asymptomatic patients were picked up by community screening using viral RNA qPCR but did not have fever or other symptoms. In subsequent statistical analysis, asymptomatic, mild, and moderate patients were grouped into the non-severe group to compare with patients with severe disease (i.e., hospitalized severe diseases and dead). According to hospital guidelines implemented at that time, interferon-based therapy was given to patients who were symptomatic, especially for those with moderate to severe disease. The therapy was composed of Interferon beta-1b 0.25mg (8 MIU) subcutaneous alt day (maximum 7 doses), up to 14 days of symptom onset. In addition, various anti-viral drugs were supplemented to interferon treatment including Ribavirin, Lopinavir, and Remdesivir. They are grouped into one single group of interferon treatment.

### 2.2 RNA extraction, reverse transcription and real-time qPCR

Total RNA was extracted from whole blood using a modified AGPC method with 1-Bromo-3-chloropropane (B9673, Sigma-Aldrich) and isopropanol (I9516, Sigma-Aldrich). The washing and elution steps followed the TRI Reagent^®^ BD instructions from the manufacturer. First-strand cDNA synthesis of the samples was performed using the PrimeScript RT reagent Kit (#RR037A, Takara Bio, Shiga, Japan). Total RNA was transcribed in the 10 μL reverse transcription system: 2 µL 5X PrimeScript Buffer (for Real-Time), 0.5 µL PrimeScript RT Enzyme Mix I, 0.5 µL Oligo dT Primer (50 μM), 2 µL Random 6 mers (100 μM) and 5 µL RNA (Up to 1 µg/µL) of the samples. The conditions of reverse transcription were 37°C in 15 minutes of incubation, followed by 85°C in 5 seconds of RT enzyme denaturation, and finally 4°C in short-term preservation until storage in the refrigerator. The reaction was performed in a C1000 Touch Thermal Cycler (Bio-Rad, Hercules, California, USA).

The synthesized cDNA was used for real-time qPCR by TB Green® Premix Ex Taq™ II (Tli RNase H Plus) kit (#RR820A, Takara Bio, Shiga, Japan). Each sample was duplicated in real-time qPCR analysis. The expression levels of target genes were calculated in terms of Ct value. The PCR conditions were pre-incubation (95°C in 30 seconds), subsequently 45 cycles of amplification (95°C in 5 seconds, 55°C in 30 seconds, and 72°C in 20 seconds), melting (95°C in 1 minute, 40°C in 1 minute and 65°C in 20 seconds), and finally the coiling (40°C in 30 seconds). The reaction was carried out in the LC480 thermal cycler (Roche, Basel, Switzerland).

### 2.3 SARS-CoV-2 infection diagnosis and viral load analysis

Viral load may be an important factor affecting the disease course and severity. Multiple samples were collected from each patient to measure viral loads and the peak viral load defined as the highest value of viral load during the course of infection was used in statistical analysis. Viral RNA was extracted from nasopharyngeal swabs using the QIAamp Viral RNA Mini Kit (Qiagen, Hilden, Germany) according to the manufacturer’s instructions. SARS-CoV-2 RNA was quantified using RT-PCR. The primer-probe set N1 (2019-nCoV_N1-F: 5’-GAC CCC AAA ATC AGC GAA AT-3’, 2019-nCoV_N1-R: 5’-TCT GGT TAC TGC CAG TTG AAT CTG-3’ and 2019-nCoV_N1-P: 5’-FAM-ACC CCG CAT TAC GTT TGG ACC-BHQ1-3’) designed by US Centers for Disease Control and Prevention (CDC) were purchased from Integrated DNA Technologies, USA. The one-step real-time RT-PCR reaction contained 5 μL of the extracted preparation, 4 μL TaqMan™ Fast Virus 1-Step Master Mix (Applied Biosystems, USA) in a final reaction volume of 20 μL. The primer and probe concentrations were 0.5 μM and 0.125 μM, respectively. The cycling conditions, 25°C for 2 min, 50°C for 15 min, 95°C for 2 min, followed by 45 cycles of 95°C for 15 s, and 55°C for 30 s, were performed with the StepOnePlus Real-Time PCR System (Applied Biosystems, USA). Samples were considered as negative if the Ct values exceeded 39.9 cycles. The Ct values of real-time RT-PCR were converted into viral RNA copies based on a standard curve prepared from 10-fold serial dilutions of known copies of a plasmid containing the full N gene (2019-nCoV_N_Positive Control, Integrated DNA Technologies, USA).

### 2.4 Data analysis of qPCR and IFN score calculation

Based on the MIQE principle, all RT-qPCR experiments include calibrators and PCR efficiency. PCR efficiency is calculated from serially diluted samples. The calibrator is a constant human cDNA sample obtained from the same healthy donor. The duplicate Ct value difference must be less than 1. The delta-delta Ct method was used for the relative quantification of transcript abundance (TA). Gene UBC and RPL31 were used as the internal housekeeping (HK) gene. The fold change (FC) for each gene (g) was calculated by an efficiency corrected ddCT method as: FCg=eg(CTg_calibratior - CTg_mean)/median[ehk(CThk_calibratior - CThk_mean)]. The median based IFN score was calculated as follows: IFN score=Median of (FC(IFIT1), FC(IFIT2), FC(IFI27), FC(IFI44L), FC(SIGLEC1)). IFN scores and FC of gene expression shown in the figures are log transformed by a base of 2 (i.e. log2 transformation). Spearman correlations between IFN score and each of the IFN genes were shown in **Supplementary Figure SF1**.

### 2.5 Public COVID-19 single-cell RNA-seq datasets

PBMC scRNA-seq datasets of COVID-19 patients were collected from two publicly available resources, namely the CZ CELLxGENE Discover platform (https://cellxgene.cziscience.com/) and the Gene Expression Omnibus (GEO) database (**Table 2**). To retrieve the relevant datasets, filters were applied using keywords ‘COVID-19’, ‘Homo sapiens’, and ‘blood PBMC’. A total of ten datasets (7, 23–31) that fulfilled the aforementioned criteria were obtained from the CELLxGENE Discover platform. Furthermore, two datasets, GSE171555 and GSE192391 (32, 33), were selected from the GEO database for downstream analysis. Subsequently, all the datasets were subjected to preprocessing steps to ensure data quality control and consistency.

**Table 2:** General information of public single cell RNA-seq datasets used in this study.

Datasets (No. of cells)	Publications	Platform	Patients	Healthy
Ahern et al. (836,148) EGAS00001005493	A blood atlas of COVID-19 defines hallmarks of disease severity and specificity (23)	10x 5’ v1; CITE-seq	100	10
Yoshida et al. (422,220) GSE168215	Local and systemic responses to SARS-CoV-2 infection in children and adults (24)	10x 5’ v1; CITE-seq	20	50
Van der Wijst et al. (600,929) GSE168453	Type I interferon autoantibodies are associated with systemic immune alterations in patients with COVID-19 (25)	10x 5’; CITE-seq	54	11
Stephenson et al. (647,366) E-MTAB-10026	Single-cell multi-omics analysis of the immune response in COVID-19 (26)	10x 3’; CITE-seq	69	35
Ren et al. (1,462,702) GSE158055	COVID-19 immune features revealed by a large-scale single-cell transcriptome atlas (27)	10x 3’ v3; 10x 5’ v2	35	25
Liu et al. (372081) GSE161918	Time-resolved systems immunology reveals a late juncture linked to fatal COVID-19 (28)	10x 5’ v1; CITE-seq	32	14
Wilk et al. (44,721) GSE150728	A single-cell atlas of the peripheral immune response in patients with severe COVID-19 (29)	Seq-Well	7	6
Lee et al. (59,572) GSE149689	Immunophenotyping of COVID-19 and influenza highlights the role of type I interferons in development of severe COVID-19 (7)	10x 3’ v3	8	4
Schulte-Schrepping et al. (90,957) EGAS00001004571	Severe COVID-19 Is Marked by a Dysregulated Myeloid Cell Compartment (30)	10x 3’ v2; 10x 3’ v3	18	21
Arunachalam (49,139) GSE155673	Systems biological assessment of immunity to mild versus severe COVID-19 infection in humans (31)	10x 3’ v3; CITE-seq	7	5
Yu et al. (424,080) GSE171555	Mucosal-associated invariant T cell responses differ by sex in COVID-19 (32)	10x 5’ v1	16	8
Amrute et al. (199,097) GSE192391	Cell specific peripheral immune responses predict survival in critical COVID-19 patients (33)	10x 5’	12	6

#### 2.5.1 Pre-processing single-cell RNA-seq data and pseudobulk preparation

The CELLxGENE collections comprise more than ten pre-processed COVID-19 single-cell RNA-seq datasets in which the cells have been well labeled. Only COVID-19 cases within 10 days of onset and healthy controls from the ten selected datasets were included in this study. The raw datasets selected from GEO (GSE171555 and GSE192391) underwent quality control, where low-quality cells or doublets were removed based on the number of expressed genes fewer than 200 or greater than 4000 or had mitochondrial counts > 25%. The remaining data were then normalized using the ‘NormalizeData()’ function from Seurat (34) and log-normalization was performed with a scale factor of 10000. Cell annotations were generated using a reference-based method with azimuth datasets (34, 35). Specifically, Seurat’s ‘FindTransferAnchors()’ and ‘MapQuery()’ functions were used to map cells to the reference azimuth dataset (i.e. PBMC multimodal data). Predicted cell types for each cell were manually validated. The labeled datasets were used to create pseudobulk data, which has been shown to improve the performance of differential expression analysis in single-cell RNA-seq data (36–38). Pseudobulk data was generated by summing the raw counts for each gene across individuals and cell types, including monocyte, B cell, CD4+ T, CD8+ T, and NK cells, resulting in five gene × individual matrices.

#### 2.5.2 Quality control for the pseudobulk datasets

To ensure data quality and reduce noise, a series of filtering steps were performed at the gene and sample levels. Firstly, if genes that were missing in any of the datasets or with a maximum log count-per-million (log CPM) value less than 5 in all the five cell-types, pseudobulk data were removed. Secondly, outlier samples (i.e. individuals in pseudobulks) were excluded based on Mahalanobis distance metrics that were computed using 19 selected housekeeping genes (39–41). Finally, for datasets with batches (e.g. Liu et al. dataset), Combat-seq (42) was applied to reduce the batch effects. These steps ensured that the data used in the downstream analysis passes quality control and had minimal technical artifacts.

#### 2.5.3 Differential expression analysis at the pseudobulk level

EdgeR (43) likelihood ratio test (LRT) was used to compare gene expression profiles between COVID-19 patients and healthy controls across the five cell types at the pseudobulk level. Prior to the analysis, the normalized library size factor was calculated for each of the quality-controlled datasets using edgeR. To visualize the effect sizes and corrected p values, bubble plots were created. Additionally, raw count gene expression levels were normalized by limma voom (44) to the log2 space for visualization purposes only. To facilitate comparisons between different cell types at the pseudobulk level, gene expressions were presented as log2 (target gene/reference gene). This simple normalization approach was consistent with our qPCR analysis and allowed for the measurement of target gene transcript abundance and comparison of expression differences between cell types. Two different housekeeping genes (UBC and RPL31) were used as reference genes.

#### 2.5.4 Comparisons between cell types stratified by disease status

To investigate the expression differences of the target IFN genes among various cell types within different disease status, we generated two pseudobulk datasets from Stephenson et al. (26) and Van der Wijst et al. (25). These datasets were chosen for their relatively balanced case-control numbers and the availability of the highest cell counts/individuals. Raw count gene expression levels were normalized by limma voom with edgeR normalized library size factors, and expressions were presented as log2 (target gene/reference gene) to enable comparability of gene expression levels among different cell types at the pseudobulk level. This simple normalization method was consistent with our qPCR analysis.

#### 2.5.5 Public peripheral blood bulk transcriptome data of COVID-19 patients

Four peripheral blood transcriptomes (i.e. bulk RNA-seq) datasets were obtained from NCBI GEO database (GSE152641, GSE157103, GSE161731 and GSE171110) (**Table 3**). For each of the datasets, low-expressed genes were removed based on the edgeR (43) filtering step with default parameters. Data was then normalized by edgeR library-size normalization and log2 transformed by limma voom (44). The ratio-based gene expression levels were calculated as log2(target gene)-log2(reference gene) as calculated in qPCR analysis. A meta-analysis of the selected IFN gene expression levels was performed between COVID-19 and healthy controls using both fixed effect models and random effect models in R packages ‘meta’, ‘rmeta’ and ‘metafor’ (49). Standardized mean differences and 95% confidence intervals were calculated using the ‘Hedges’ method for each of the datasets and meta-analysis.

**Table 3:** General information of public blood bulk RNA-seq datasets used in this study.

Datasets	Publications	Platform	Patients	Healthy
GSE152641	Transcriptomic similarities and differences in host response between SARS-CoV-2 and other viral infections (45)	GPL24676 Illumina NovaSeq 6000	62	24
GSE157103	Large-Scale Multi-omic Analysis of COVID-19 Severity (46)	GPL24676 Illumina NovaSeq 6000	100	26
GSE161731	Dysregulated transcriptional responses to SARS-CoV-2 in the periphery (47)	GPL24676 Illumina NovaSeq 6000	77	16
GSE171110	CD177, a specific marker of neutrophil activation, is associated with coronavirus disease 2019 severity and death (48)	GPL16791 Illumina HiSeq 2500	44	10

A total of 4 public blood bulk RNA-seq datasets for COVID-19 were used in this study.

### 2.6 Statistical analysis

Statistical analyses were performed using the R statistical package (R 4.2.1). Clinical variables, including sample size, age, sex, smoking status, day from onset, whether received IFN treatment, C-reactive protein (CRP) level, peak viral load, and various cell counts, percentages, and ratios, were summarized as the basic characteristics and stratified by disease severity. Group differences were assessed using t-test for normally distributed continuous variables, the Mann Whitney U test for continuous variables with skewed distributions, and the chi-square test for categorical variables. ROC curves were used to evaluate sensitivity and specificity for every possible cut-off for the potential biomarkers in bulk RNA-seq data. For RNA-seq data, gene counts were normalized using the TMM method (43). In the analysis of single-cell RNA-Seq (scRNAseq), we used the Kruskal-Wallis test to determine the expression differences among different cell types, stratified by disease status.

Correlation analysis between IFN score and continuous clinical variables was performed by Spearman correlation. Associations between IFN score and categorical clinical variables were tested by Wilcoxon Rank sum test (two categories) or Kruskal-Wallis test (more than two categories) with pairwise comparisons. The correlations between IFN score and each of the single gene were performed by Spearman correlation and were visualized using R package ‘PerformanceAnalytics’. Other plots were created by ‘ggpubr’. P values < 0.05 were considered significant.

## 3 Results

### 3.1 Characteristics of COVID-19 patients

The COVID-19 patients (n = 81, 48.15% females) included in this study had an average age of 39.95 ± 24.72 years. Patients were divided into two groups based on their severity and 13 (16%) of them were classified as severe (**Table 1**). Similar to other clinical reports, COVID-19 severity was positively correlated with age (p<0.001) and men were likely to get severe disease (p=0.02). Furthermore, all the severe cases were current smokers. Elevated CRP levels were associated with COVID-19 severity (p=0.01). Additionally, severe cases were associated with decreased complete blood count (CBC) (p=0.002), and lymphocytes % count (p<0.001) and thus a higher neutrophil to lymphocyte ratio (p<0.001).

Although there were 105 patients in the cohort, 24 of them did not have blood sample available for analysis during the first 10 days after disease onset. Another 6 patients had a poor RNA yield which may be due to sample degradation. Finally, only 75 patients had blood sample available for ISGs expression qPCR analysis and determination of IFN score.

### 3.2 qPCR IFN scores correlate with percentages of lymphocytes but showed no obvious difference between severity groups

**Figure 2:**
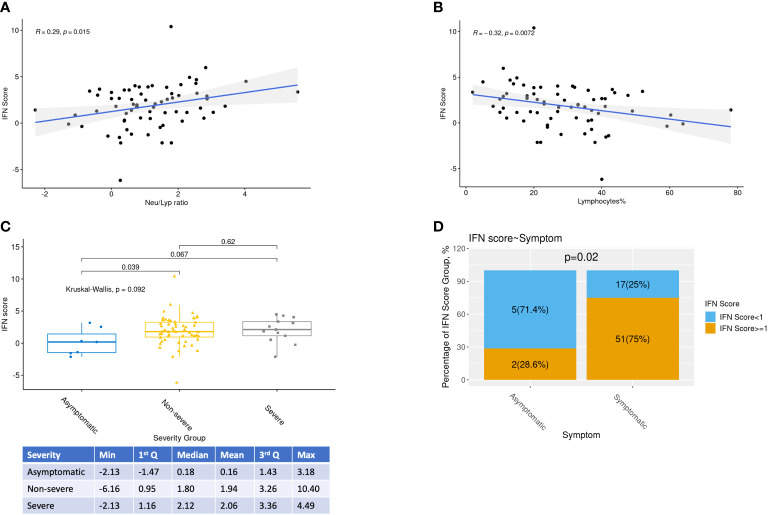
Relationships between IFN scores and selected clinical variables. Relationships between IFN scores, Neutrophil-Lymphocyte ratio, percentage of Lymphocytes, COVID-19 severity and day from onset were highlighted. (A,B) Relationships between IFN scores, Neutrophil-Lymphocyte ratio and percentage of Lymphocytes were investigated by Spearman correlations with R and p values shown. (C) Kruskal-Wallis test and pairwise Rank Sum tests were performed to study the association between IFN score and COVID-19 severity. (D) Proportion of low interferon responder (log2 IFN score <1) among patients with or without symptom

Associations between qPCR IFN scores and all clinical variables were examined (**Figure 2** and **Supplementary Figure SF2**). As expected, a positive correlation between IFN score and neutrophil-to-lymphocyte ratio in blood was observed (p=0.02) (**Figure 2A**), while the percentage of lymphocytes was negatively associated with IFN score (p=0.007) (**Figure 2B**). Furthermore, asymptomatic patients had a lower IFN score compared to both symptomatic groups mild and severe group (**Figure 2C**). For the whole cohort, IFN score of Day3-6 was significantly higher than Day0-2. However, after separating into subgroups of patients with or without interferon treatment, this difference was no longer significant.

When the routine clinical laboratory results were analyzed, WBC and CBC were negatively associated with IFN scores, whereas higher peak viral load, and percentages of monocytes and neutrophils were correlated with higher IFN scores (**Supplementary Figure SF2A**). On the other hand, IFN scores was not associated to smoking status nor age (**Supplementary Figures SF2B, D**). Although asymptomatic patients had borderline significant lower IFN score than non-severe symptomatic patients (p=0.04), there was no apparent difference in IFN scores between the 2 groups of symptomatic patients (severe vs non-severe symptomatic patients) (**Figure 2C**).

Next, the IFN-based treatment effect on IFN score was explored, stratified by different days from onset. Interestingly, there was no significant difference in IFN scores between the treatment and no treatment groups in all three time periods sample groups (p>0.2), indicating that IFN-based treatment might not add any substantial change in the endogenous IFN response.

### 3.3 Characteristics of low interferon responder COVID patients

**Figure 3:**
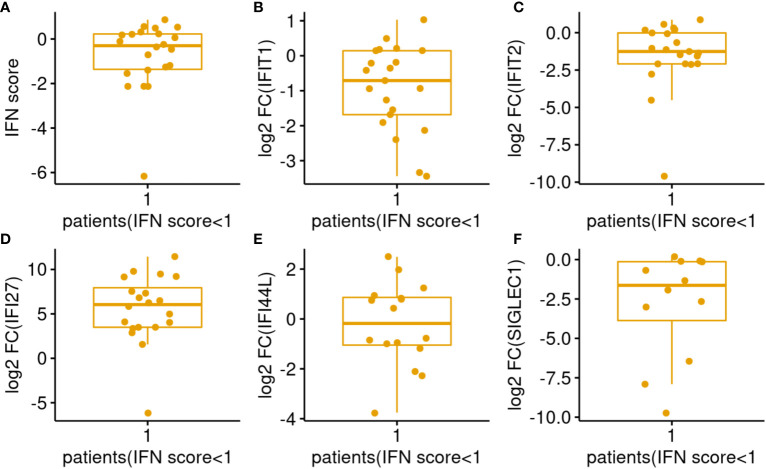
Distributions of IFN score/genes among COVID-19 patients with IFN score <1. **(A–F)** Represents IFN score, ISG IFIT1, IFIT2, IFI27, IFI44L, SIGLEC1 respectively.

Interferon score represents the median of the elevation fold changes of 5 ISG. In the figures, this score is expressed in log2 scale. Therefore, a value of 1 represents 2 folds activation for the median ISG which signify a significant IFN elevation and it also indicates that there are 2 other genes activated by more than 2 folds. Therefore, patients with log2 IFN score less than 1 were labelled as low responder. 22 out of 75 (29%) patients were low responders (**Figure 2D**). Among the low responders, we looked into the expression level of individual ISGs (**Figures 3B–F**). Although the median log2 expression of the 5 ISGs were below 1, two ISGs showed sign of activation. They were IFI27 and IFI44L (**Figures 3C, D**). IFI27 (**Figure 3D**) showed the most notable activation and the median activation was above 5 even among these low responders. So, the low IF score (**Figure 3A**) was due to lack of activation of the 3 other ISGs (IFIT1, IFIT2, SIGLEC1).

As shown in **Table 4A**, majority of low interferon responders were asymptomatic patients (chi square test, p<0.05). On the other hand, IFN score did not predict disease severity among symptomatic patients (**Table 4B**).

**Table 4:** Shows the disease characteristics of the 2 responder groups.

(A) Patients with asymptomatic COVID-19 disease or symptoms with different IFN response.			
	Asymptomatic COVID-19	COVID-19	
Low responders (29%)	5	17	
Adequate responders (71%)	2	51	
(B) Among symptomatic COVID-19 patients, IFN score did not predict severity.			
	Mild disease	Severe disease	Total
Low responders	14	3	17
Adequate responders	41	10	51

P < 0.05 by Fisher exact test.

Not significant by Fisher exact test.

### 3.4 Meta-analysis of ISG expression in bulk RNA sequencing of peripheral blood samples

**Figure 4:**
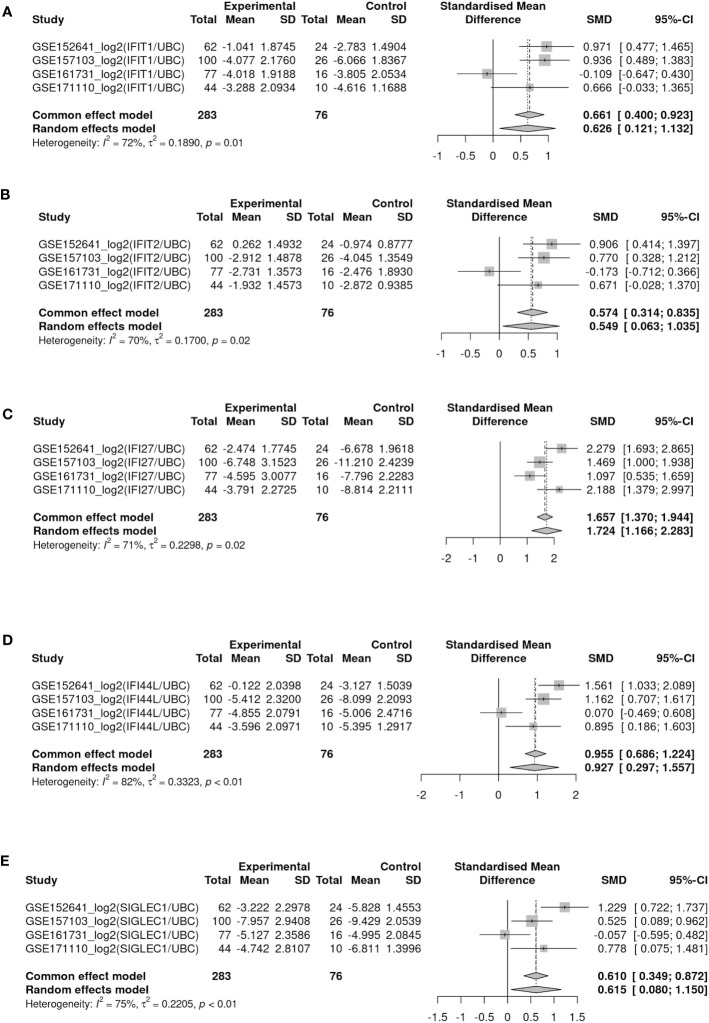
Forest plots showed meta-analysis of target IFN gene expression levels (log-ratio using UBC as reference gene) between COVID-19 and healthy control among four blood bulk RNA-seq datasets (GSE152641, GSE157103, GSE161731 and GSE171110). **(A–E)** Represents gene IFIT1, IFIT2, IFI27, IFI44L, SIGLEC1 respectively.

Patients summary of the 4 peripheral blood bulk gene expression datasets were given in **Table 3**. The effect size of ISG activation by SAR-CoV-2 was analyzed by standardized mean difference (SMD) across the 4 datasets. ISG expression was normalized by a housekeeping gene (e.g. UBC as results shown in **Figures 4A–E**). Among the IFN genes analyzed by meta-analysis, IFI27 was the most highly activated (**Figure 4C**). The SMD between COVID patients and control was 1.72 (95% confidence interval (CI): 1.16-2.28). IFI44L was the next highly activated gene SMD at 0.93 (95% CI: 0.29-1.56) (**Figure 4D**). Similar finding was observed when the data was normalized by another housekeeping gene, RPL31 (**Supplementary Figure SF3**) As the data were log-transformed before analysis, such magnitude of increase represented 2 to more than 4 folds increase in expression of these interferon genes in the peripheral blood samples.

### 3.5 Selected IFN genes are highly expressed in COVID-19 monocytes

**Figure 5:**
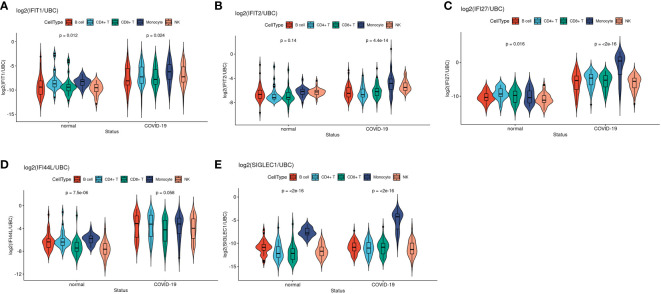
Violin plots showed comparisons of target IFN gene expression levels between disease status among five main cell types at pseudobulk level in EMTAB-10026 single cell RNA-seq dataset. **(A–E)** Represents gene IFIT1, IFIT2, IFI27, IFI44L, SIGLEC1 respectively.

We next studied the selected IFN gene expressions across five main leukocyte cell types in single cell RNAseq datasets (i.e. monocyte, B cell, CD4+ T cell, CD8+ T cell and NK cell) in COVID-19 cases and healthy controls at single cell and pseudobulk level (sum aggregation of single-cell RNA-seq data). **Figure 5** are the violin plots of the 5 ISGs of COVID-19 patients compared to healthy controls. From the scRNAseq results, it was evident that both IFI27 and SIGLEC1 were ISG predominantly expressed by monocytes. IFI27 is expressed across cell types at baseline but it was only activated in monocyte after infection (**Figure 5C**). On the other hand, both baseline expression and post-infection activation were predominant in monocytes among leukocyte cell types in PBMC. IFIT1 and IFIT2 are well described neutrophil expressed ISG and their expression are most prominent in mature neutrophil after interferon stimulation (G5) (50–53) (**Figures 5A, B**). In differential expression analysis, four out of the five selected ISGs (IFIT1, IFI27, IFI44L and SIGLEC1) showed higher expression in COVID-19 than healthy control (**Figures 5A–E**). Among healthy subjects, assumed as the baseline expression when expressed as pseudobulk data, four (IFIT1, IFIT2, IFI27 and IFI44L) out of the five selected IFN genes were universally expressed among the 5 major cell types, while SIGLEC1 was preferentially expressed by monocytes (**Figure 5E**). These gene expression levels were transformed to target/reference gene log ratios for better visualization (See Methods for details). Two different reference genes (UBC: shown in **Figure 5**; RPL31: shown in **Supplementary Figure SF4**) were used and both showed similar results.

Taken together, the larger effect size between COVID-19 and healthy controls as well as the higher expression levels in monocytes suggested that IFI27 and SIGLEC1 is a monocyte-specific biomarker with slightly different property in response to viral infection. Although IFN genes such as IFI27 and IFI44L are also highly expressed in various cell types in COVID-19 samples, monocytes contributed the most among PBMCs, suggesting its active involvement in interferon response.

## 4 Discussion

Soon after the COVID-19 outbreak, it became widely accepted that the SARS-CoV-2 virus is capable of suppressing our innate immunity by impairing our interferon response (5). An impaired interferon response had been described by studies that investigated interferon pathway in COVID patients mainly focused on respiratory tissue IFN responses, such as lung tissue (54) and BALF (9). However, the ability of the virion to antagonist interferon pathway was only observed in specific *in-vitro* experiments and the ability of viral proteins to inhibit IFN responses differed among studies (55). It is not sure to what extent the inhibitory effects were due to overexpression of the viral protein in ectopic subcellular locations (56).

Interferon stimulated gene expression represents an unbiased whole-body response or bioassay of the presence and the scale of production of various interferons. Therefore, interferon scores are more clinically relevant biomarker of interferon activation. If the SARS-CoV-2 is inhibiting interferon response significantly at the host level, it should be reflected by poor interferon scores among the majority of COVID-19 patients. In fact, early studies reported interferon score was low in COVID-19, even among patients with severe and critical disease (5). Subsequently, an insufficient interferon response was also associated with adverse disease outcomes (2, 5, 57). In addition, patients with inherited interferon defects got severe COVID-19 disease. These findings would suggest that boosting the interferon pathway with exogeneous interferon would be helpful. However, the WHO solidarity trial concluded that none of the 4 repurposing drugs (including a treatment limb of a 6-day course of interferon beta-1a) could prevent severe disease in COVID-19 patients (58). The results contradicted the notion that COVID-19 patients had impaired interferon responses.

Here, we tried to tackle these contradictory findings by a systemic analysis on ISGs expression in blood as a bioassay of the interferon response of the human host. Many early studies either did not specify the days after disease onset when blood or tissue samples were collected for study or collected samples later than the first week. For example, Hadjadj et al. collected samples after a median of 10 days after disease onset (5). Our study limited the sample collection period to the first 10 days of infection (i.e. the early stage after infection) and our finding suggested that most patients, in fact, mounted an adequate interferon response and it can be reflected by IFN score. Then, the IFN score declined naturally as in other viral infections. It is now documented that the interferon response and score are only activated during the very early phase of viral infection and will largely be subsided at the end of the first week (55, 56, 59).Therefore our data further confirmed the importance of the timeframe for sample collection and examination of IFN genes Similarly, contradictory results of treatment with various exogenous interferon in different trials could be due to difference in the outcome measures and timing of the interferon administration. Whether interferon is administered early (within the first few days after onset) or late may result in completely different outcomes. This is a conclusion made by Jhuti et al. after reviewing the clinical trials including interferon administration before the TOGETHER study reported in 2023 (16, 60). Brzoska et al. also arrived at the same conclusion after reviewing four major clinical phase III trials with interferons (59). Our study is quite unique that we have the chance to analyze blood samples collected within the first 10 days after onset. Therefore, we provided a more realistic picture of interferon activation in COVID-19 patients. And we showed that most COVID-19 patients did mount an appropriate interferon response after SAR-CoV-2 infection.

However, 29% of patients (22 out of 75 patients in our cohort) whose interferon scores were less than 2 times of the healthy control. Five of them (23%) were asymptomatic patients. Most of these low interferon responders experienced only mild diseases that did not require hospital admission. Only 3 of them (14%) had a severe disease. Statistical analysis suggested that low interferon responders were more common among the asymptomatic patients (P value < 0.05 by Fisher Exact test). But the comparison of disease severity among symptomatic patients suggested that low interferon responders did not differ from other patients with good interferon activation. They only had partial ISGs activation confining to only 1 out of the 5 ISGs examined. In other words, low interferon responders are more likely to be asymptomatic patients but this status does not predict disease severity. It is not well understood why some patients were asymptomatic. A hypothesis may be drawn from the latest genome-wide association study report. Pairo-Castineira et al. reported the latest GWAS on COVID-19 so far (61). Many genes in the interferon pathway are found among the top hits in the largest GWAS up-to-day. For example, a SNP on the JAK1 gene (rs12046291) was associated with critical COVID-19. It may suggest that some alleles in JAK1 might affect the extent of JAK1 activation by interferon. However, this is still pending experimental validation. Other associated SNPs are found in genes like IFNAR2, OAS1 and IFNA10.

Concerning the hematological cell-type that accounts for the ISG expression after interferon activation, single cell RNA sequencing provided the information. While most of the ISG are expressed by most cell types, IFI27 is more specific to monocytes. It has a higher expression in monocytes in COVID-19 samples and the activation is also more intense. Nonetheless, it keeps consistent expression levels among all the selected cell types in normal samples. Additionally, it is worth noting that the gene SIGLEC1 is apparently specific to monocytes in both COVID-19 and normal samples. Regardless of the disease status, it shows consistently higher expression levels in monocytes than the other cell types. Interestingly, larger expression differences between COVID-19 and normal samples are also presented in monocytes with higher statistical significance than any other selected cell types. This phenomenon suggests that certain specific gene activities uniquely or dominantly take place within monocytes in COVID-19 samples and could be investigated in depth. In the future, drug trials with interferon therapy should be targeting at this subpopulation of patients with a partial ISGs activation. While giving interferon injections to all COVID-19 patients might not be helpful to prevent severe disease, a more targeted approach to a subgroup of patients may be more effective.

There has been a lack of data on ISGs in clinical whole blood sample and many researchers suggested that IFN response in COVID-19 patients was inadequate. We detected the ISGs and calculated the IFN score to reflect the systemic IFN response in the COVID-19 patients. Compared to healthy controls, the IFN score of COVID-19 patients was increased on average by 20.94 folds. Therefore, most COVID-19 patients did not have inadequate IFN responses, which was reported recently in other studies (11). Contrary to other studies suggesting that COVID-19 patients have insufficient IFN response, there was a marked interferon response of COVID-19 patients in our cohort, particularly during the early phase of COVID-19 (6, 54). To investigate the reasons for these contrasting results, we also investigated the effect of sampling times. If the blood samples were collected late (e.g. one week after fever onset), ISGs was lowered or returned to normal levels (28). This was also found in our cohort, peripheral blood ISGs levels changed as early as during the first few days after onset of COVID-19 infection. In common with more recent reports (28, 30, 62), attention about the sampling time is strongly recommended when describing and interpreting the expression level of ISGs in COVID-19 patients. For example, samples were collected at a median of 10 days after disease onset in Hadjadj et al. (5), the late-collected sample set might explain why interferon activity appeared to be impaired. As for the association with severity, one study reported impaired IFN-I activity and decreased ISGs in blood in severe COVID-19 patients (5). While in our cohort, association was not found between ISGs and disease severity.

## 5 Conclusion

**1.** In a cohort of vaccine-naive patients, we showed an early and significant systemic interferon response in the majority (71%) of patients after COVID-19 infection. It suggests that COVID-19 induces a sufficient endogenous IFN response.

**2.** IFI27 and SIGLEC1 were predominately expressed by monocyte and were most intensely activated after SAR-CoV-2 infection. These two monocyte-predominant expressing ISGs are useful biomarkers to monitor IFN response in monocyte in peripheral blood.

**3.** Low interferon responders (as defined by the IFN score) are common among asymptomatic patients. Even though many ISGs (such as IFIT1) were not raised, the monocyte-predominant ISG, IFI27 was activated in most of them. It is not known why IFN response was confined to monocytes but not extended to other cell types in peripheral blood. This patient subgroup may benefit from exogenous interferon treatment. However, the overall IFN score and individual ISG levels did not predict disease severity.

## Data availability statement

The datasets presented in this study can be found in online repositories. The names of the repository/repositories and accession number(s) can be found in the article/**Supplementary Material**.

## Ethics statement

The studies involving humans were approved by The Joint Chinese University of Hong Kong – New Territories East Cluster Clinical Research Ethics Committee. The studies were conducted in accordance with the local legislation and institutional requirements. The participants provided their written informed consent to participate in this study.

## Author contributions

BH: Writing – original draft, Investigation. JH: Data curation, Investigation, Writing – original draft, Validation. NC: Formal analysis, Writing – original draft. ZC: Conceptualization, Writing – review & editing. GL: Conceptualization, Writing – review & editing. LL: Supervision, Writing – review & editing. MK: Investigation, Writing – review & editing. JW: Investigation, Writing – review & editing, Validation. PM: Investigation, Writing – review & editing. JL: Investigation, Writing – review & editing. IL: Investigation, Writing – review & editing. RN: Conceptualization, Writing – review & editing. XW: Methodology, Writing – review & editing. RG: Validation, Writing – review & editing. DH: Conceptualization, Writing – review & editing. SM: Conceptualization, Writing – original draft. PC: Funding acquisition, Supervision, Writing – review & editing. NT: Conceptualization, Supervision, Writing – review & editing.
