# Supplementary material for: Interferon response and profiling of interferon response genes in peripheral blood of vaccine-naive COVID-19 patients
Source: Front Immunol. 2024 Jan 10;14:1315602. doi: 10.3389/fimmu.2023.1315602 (PMC10806211; doi:10.3389/fimmu.2023.1315602)
Supplement: Supplementary file 1 [file DataSheet_1.pdf]

Supplemental Figure 1. Correlations between median-based IFN scores and each of the IFN genes.

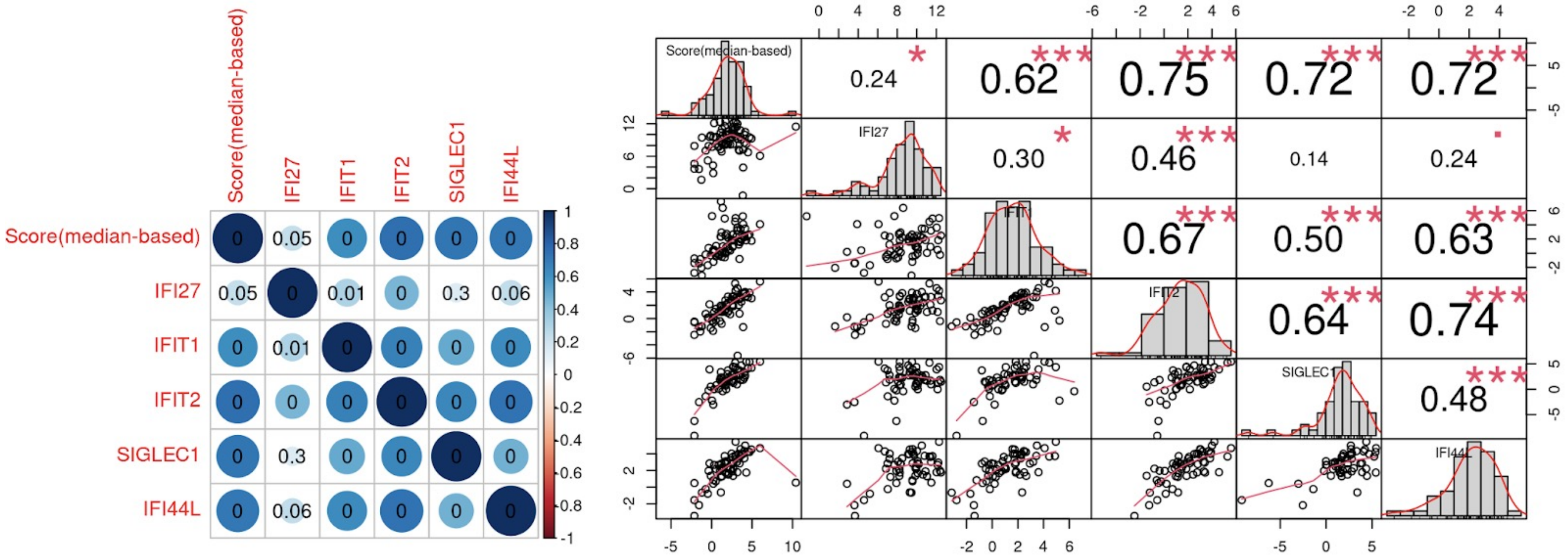

Supplemental Figure 2. Relationships between IFN scores and selected clinical variables, (A) Blood cell components; (B) smoking status; (C) day of onset; (D) age.

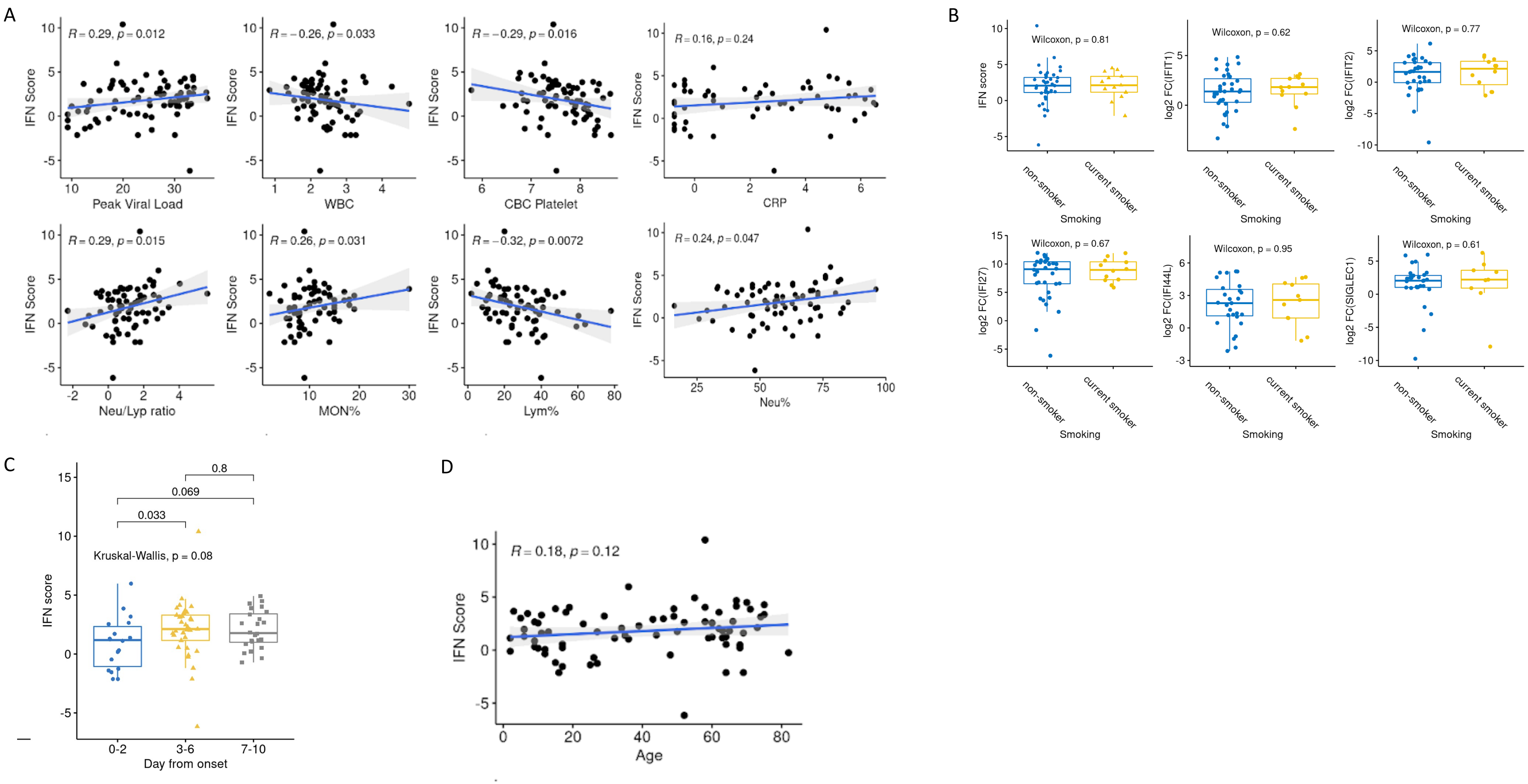

Supplemental Figure 3. Violin plots showed comparisons of target IFN gene expression levels between disease status among five main cell types at pseudobulk level in GSE168453 single cell RNA-seq dataset. (A) UBC as housekeeping gene; (B) RPL31 as housekeeping gene.

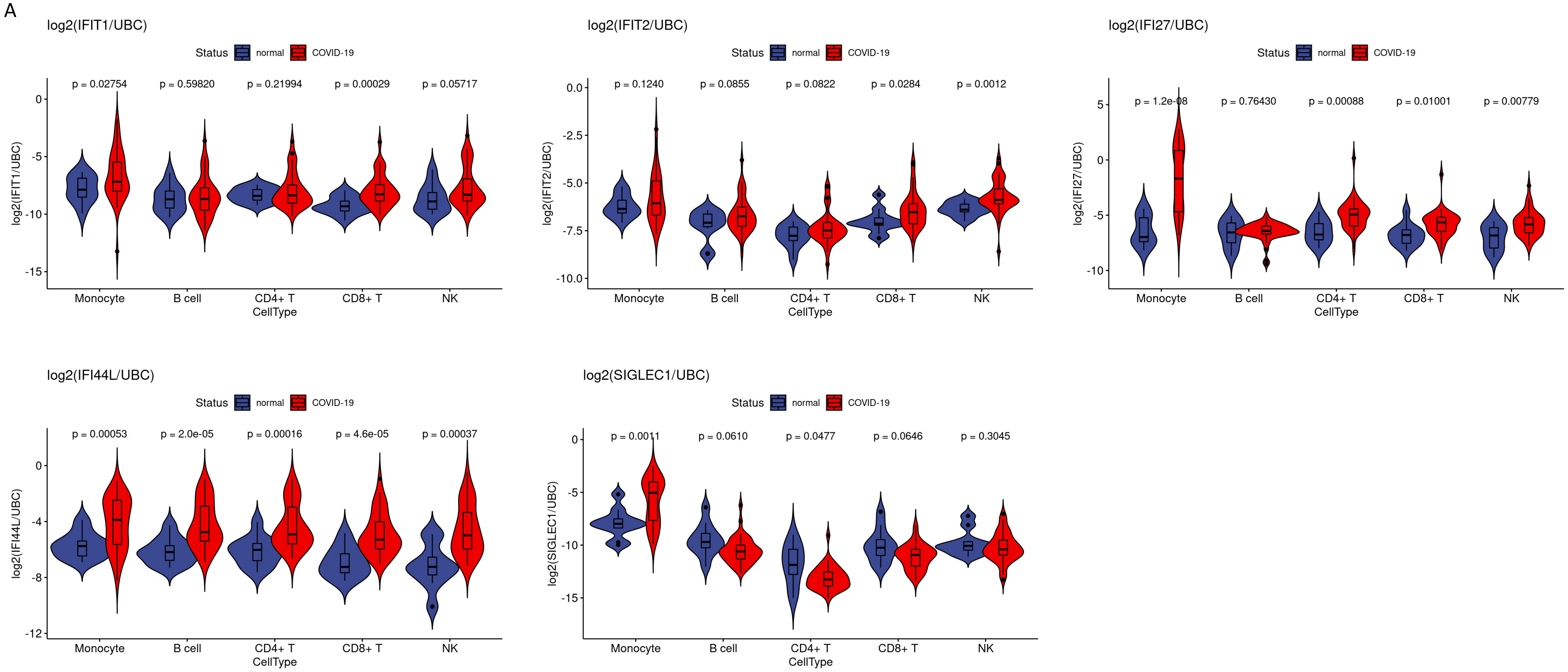

B

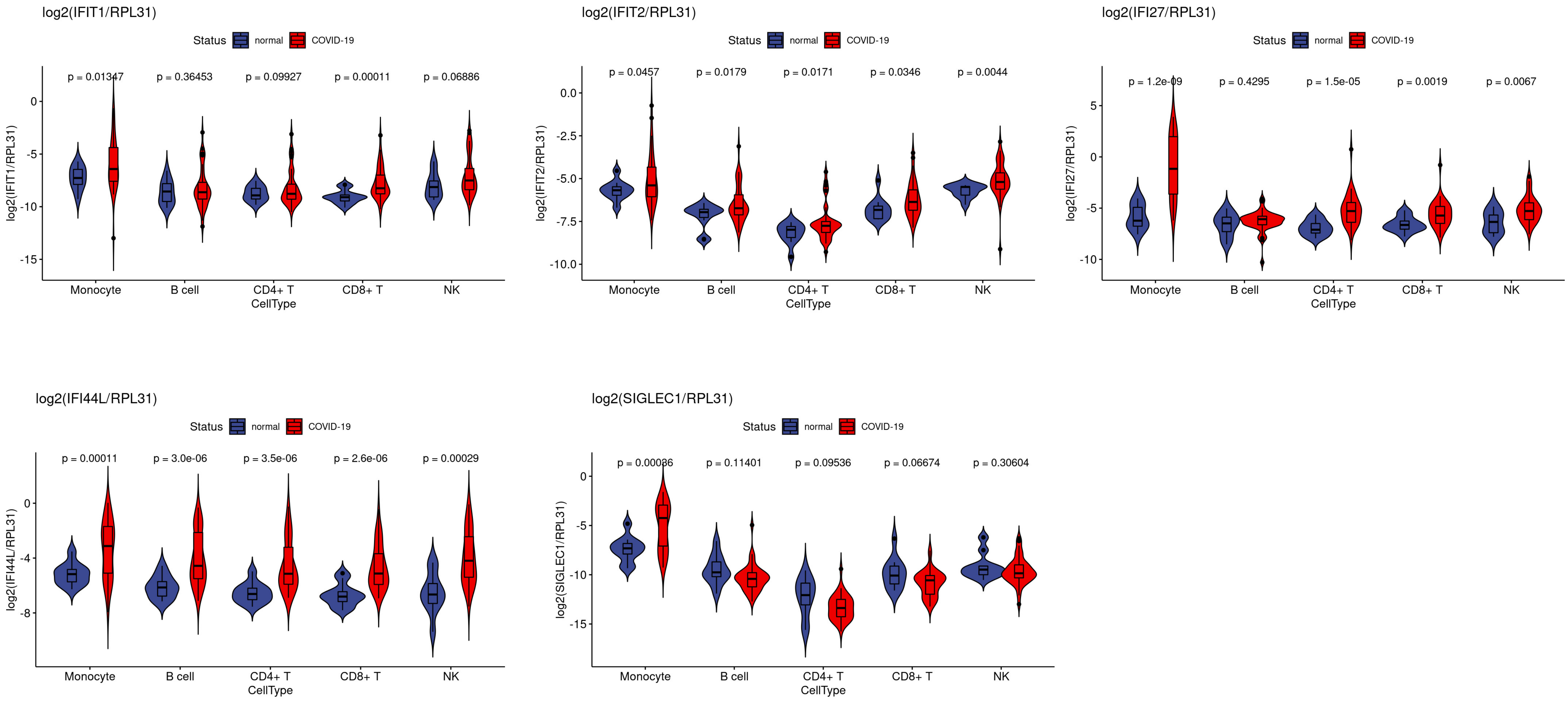
log2(IFIT1/RPL31)
Status normal COVID-19
p = 0.01347
p = 0.36453
p = 0.09927
p = 0.00011
p = 0.06886
log2(IFIT1/RPL31)
0
-5
-10
-15
Monocyte
B cell
CD4+ T
CD8+ T
NK
CellType
log2(IFIT2/RPL31)
Status normal COVID-19
p = 0.0457
p = 0.0179
p = 0.0171
p = 0.0346
p = 0.0044
log2(IFIT2/RPL31)
0.0
-2.5
-5.0
-7.5
-10.0
Monocyte
B cell
CD4+ T
CD8+ T
NK
CellType
log2(IFI27/RPL31)
Status normal COVID-19
p = 1.2e-09
p = 0.4295
p = 1.5e-05
p = 0.0019
p = 0.0067
log2(IFI27/RPL31)
5
0
-5
-10
Monocyte
B cell
CD4+ T
CD8+ T
NK
CellType
log2(IFI44L/RPL31)
Status normal COVID-19
p = 0.00011
p = 3.0e-06
p = 3.5e-06
p = 2.6e-06
p = 0.00029
log2(IFI44L/RPL31)
0
-4
-8
Monocyte
B cell
CD4+ T
CD8+ T
NK
CellType
log2(SIGLEC1/RPL31)
Status normal COVID-19
p = 0.00036
p = 0.11401
p = 0.09536
p = 0.06674
p = 0.30604
log2(SIGLEC1/RPL31)
0
-5
-10
-15
Monocyte
B cell
CD4+ T
CD8+ T
NK
CellType

Supplemental Figure 4. Forest plots showed meta-analysis of target IFN gene expression levels (log-ratio using RPL31 as reference gene) between COVID-19 and healthy control among five bulk RNA-seq datasets (GSE152641, GSE157103, GSE161731 and GSE171110).

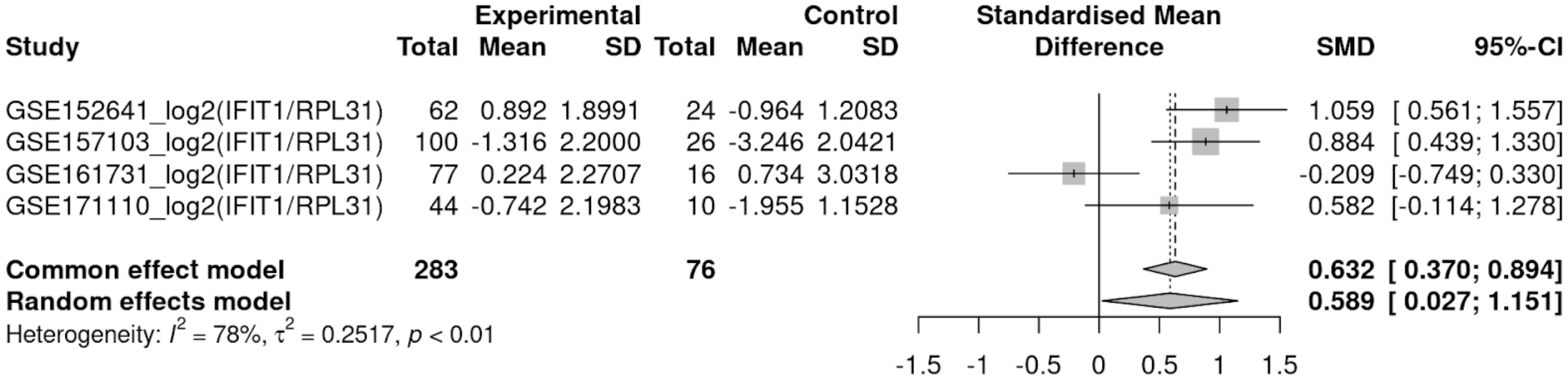

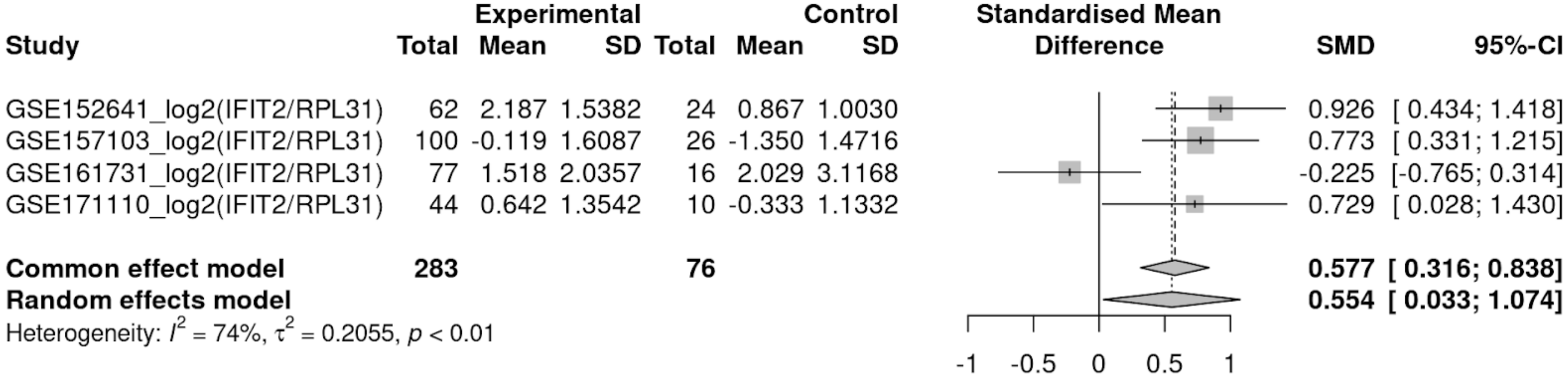

Study	Experimental Total	Experimental Mean	Experimental SD	Control Total	Control Mean	Control SD	SMD	95%-CI
GSE152641_log2(IFI27/RPL31)	62	-0.602	1.8249	24	-4.701	1.7634	2.247	[1.664; 2.830]
GSE157103_log2(IFI27/RPL31)	100	-4.015	3.1788	26	-8.285	2.3796	1.399	[0.933; 1.864]
GSE161731_log2(IFI27/RPL31)	77	-0.281	3.3392	16	-3.603	2.4011	1.028	[0.469; 1.588]
GSE171110_log2(IFI27/RPL31)	44	-1.218	2.3383	10	-6.270	2.2041	2.150	[1.345; 2.955]
**Common effect model**	**283**			**76**			**1.600**	**[1.314; 1.885]**
**Random effects model**							**1.673**	**[1.095; 2.250]**

Heterogeneity: $I^2 = 73\%$, $\tau^2 = 0.2529$, $p = 0.01$

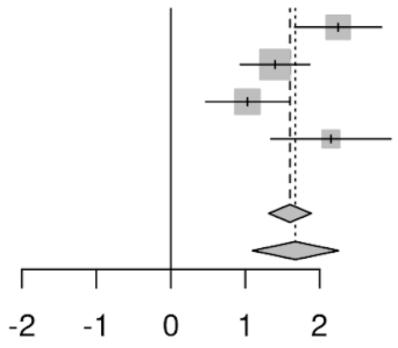

Study	Experimental Total	Experimental Mean	Experimental SD	Control Total	Control Mean	Control SD	SMD	95%-CI
GSE152641_log2(IFI44L/RPL31)	62	1.683	2.0874	24	-0.976	1.5299	1.351	[0.836; 1.865]
GSE157103_log2(IFI44L/RPL31)	100	-2.653	2.4031	26	-5.277	2.4008	1.086	[0.633; 1.538]
GSE161731_log2(IFI44L/RPL31)	77	-0.478	2.6918	16	-1.115	3.1779	0.227	[-0.312; 0.767]
GSE171110_log2(IFI44L/RPL31)	44	-1.024	2.0378	10	-2.846	1.5517	0.915	[0.206; 1.625]
**Common effect model**	**283**			**76**			**0.923**	**[0.657; 1.190]**
**Random effects model**							**0.902**	**[0.418; 1.386]**

Heterogeneity: $I^2 = 69\%$, $\tau^2 = 0.1649$, $p = 0.02$

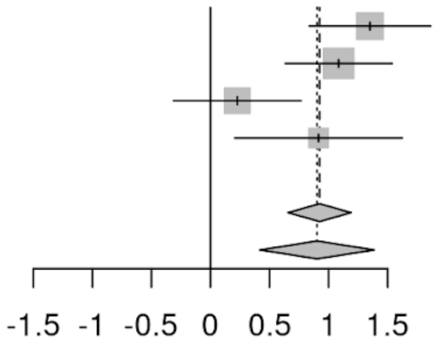

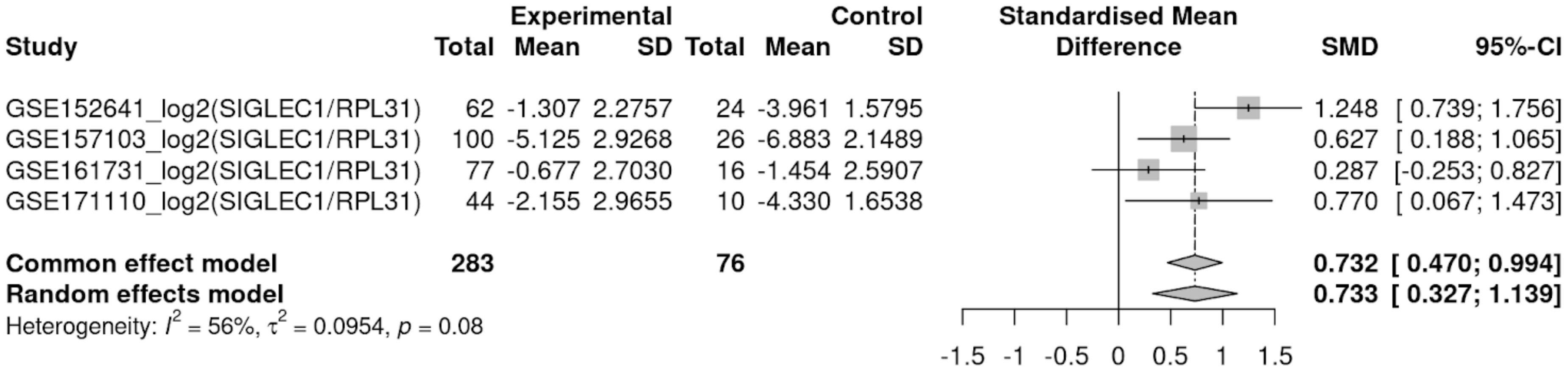

Study
Experimental
Total Mean SD
Control
Total Mean SD
Standardised Mean Difference
SMD 95%-CI
GSE152641_log2(SIGLEC1/RPL31) 62 -1.307 2.2757 24 -3.961 1.5795 1.248 [0.739; 1.756]
GSE157103_log2(SIGLEC1/RPL31) 100 -5.125 2.9268 26 -6.883 2.1489 0.627 [0.188; 1.065]
GSE161731_log2(SIGLEC1/RPL31) 77 -0.677 2.7030 16 -1.454 2.5907 0.287 [-0.253; 0.827]
GSE171110_log2(SIGLEC1/RPL31) 44 -2.155 2.9655 10 -4.330 1.6538 0.770 [0.067; 1.473]
Common effect model 283 76 0.732 [0.470; 0.994]
Random effects model 0.733 [0.327; 1.139]
Heterogeneity: $I^2 = 56\%$, $\tau^2 = 0.0954$, $p = 0.08$
-1.5 -1 -0.5 0 0.5 1 1.5
